# In Vitro Skin Delivery of Griseofulvin by Layer-by-Layer Nanocoated Emulsions Stabilized by Whey Protein and Polysaccharides

**DOI:** 10.3390/pharmaceutics14030554

**Published:** 2022-03-02

**Authors:** Daniel P. Otto, Anja Otto, Melgardt M. de Villiers

**Affiliations:** 1 Research Focus Area for Chemical Resource Beneficiation, Faculty of Natural and Agricultural Sciences, North-West University, Potchefstroom 2531, South Africa; 2 School of Pharmacy, Pharmaceutical Sciences Division, University of Wisconsin-Madison, 777 Highland Ave, Madison, WI 53705, USA; anja_otto@hotmail.de (A.O.); melgardt.devilliers@wisc.edu (M.M.d.V.)

**Keywords:** layer-by-layer, skin, emulsions, whey protein, polysaccharide

## Abstract

Griseofulvin is a poorly water-soluble drug administered orally to treat topical fungal infections of the skin and hair. However, oral administration leads to poor and unpredictable drug pharmacokinetics. Additionally, griseofulvin is unstable in the presence of light. A layer-by-layer (LbL) nanocoating approach was employed to curb these shortcomings by stabilizing emulsions, lyophilized emulsions, and reconstituted emulsions with a layer each of whey protein, and either hyaluronic acid, amylopectin, or alginic acid, which captured the drug. The coating materials are biological, environmentally benign, and plentiful. Photostability studies indicated that the LbL particles afforded 6 h of protection of the topical application. In vitro absorption studies showed that griseofulvin concentrated preferentially in the stratum corneum, with virtually no transdermal delivery. Therefore, LbL-nanocoated emulsions, lyophilized particles, and reconstituted lyophilized emulsions can produce a viable topical delivery system to treat superficial fungal infections.

## 1. Introduction

Most research has focused on the formulation and evaluation of traditional emulsions containing an oil and aqueous phase. The fine oil droplets can be dispersed in the aqueous phase, known as oil-in-water emulsions (o/w). However, these emulsions are prone to instabilities such as irreversible coalescences of the dispersed droplets [1]. The possibility of hydrolysis of the active ingredients and oxidation of the oil phase in these emulsions may also be of concern.

Previous studies [2,3] reported that the charge of the droplet had shown a significant role in the delivery of the active ingredients into or through the skin. Skin permeation was also influenced by the emulsifier ratio and, for ethoxylate polymers, by the hydrophilic–lipophilic balance value (HLB) [4,5]. Additionally, the hydrophilic chain length of non-ionic surfactants can contribute to the dermal and transdermal delivery of active ingredients from emulsions [6,7,8]. Emulsions can also be stabilized with solid particles, resulting in Pickering emulsions, showing increased dermal and transdermal delivery compared to surfactant-stabilized emulsions [2,9].

Recently, renewably sourced biopolymers have garnered significant investigation into their ability to stabilize emulsions. These polymers have the advantage of being hypoallergenic, biocompatible, and biodegradable [10,11,12,13]. By evaporating the aqueous phase from mainly o/w emulsions using spray-drying or freeze-drying, dry emulsions could be produced from biopolymer dispersions [14]. Dried emulsions increased the photostability of drugs and increased the bioavailability of poorly soluble active ingredients [15,16,17,18].

Using the LbL technique, transdermal emulsions can be produced by including whey protein on its own or in combination with *κ*-carrageenan or chitosan as an emulsifier [19,20]. LbL is the process by which successive coating of a substrate takes place with polyelectrolytes of opposite charge [21,22,23]. Whey proteins are derived from milk during the cheese-making process [11] and comprise two main ingredients, namely α-lactalbumin and β-lactoglobulin [24,25]. Whey proteins are marketed as food supplements, although their claim of health improvement is disputed [26]. Heating the whey proteins above 80 °C denatures the proteins, leading to a very viscous aqueous dispersion once dissolved. The high viscosity of the dispersion improves the stability of the resultant emulsions and may find higher-value utilization, such as in drug delivery systems [13,24].

It has been reported [27] that hyaluronic acid, a naturally occurring polymer composed of unbranched repeating units of glucuronic acid and *N*-acetyl-glucosamine, increased the diffusion and epidermal skin localization and retention [28]. Hyaluronic acid also consists of penetration-enhancing properties that lead to relatively high concentrations of active ingredients being carried through the epidermis into the dermis rapidly [29].

Griseofulvin, an antifungal drug, was chosen as the model active ingredient. Griseofulvin shows poor and erratic oral bioavailability [30]. Griseofulvin is mainly administered orally and eventually reaches the skin and hair to treat topical fungal infections [31]. However, topical administration of griseofulvin ethosomes [32] enhanced the skin delivery of griseofulvin to the lipophilic stratum corneum layer. This finding implies that oral administration of the drug is not optimal.

In this study, we report the (i) successful employment of LbL to produce biopolymer-nanocoated emulsions and dry emulsions as delivery vehicles of griseofulvin; (ii) the release of griseofulvin from the formulations and (iii) the in vitro (trans-)dermal absorption of griseofulvin from these formulations in excised human skin. The findings show that a topical LbL-nanocoated delivery system could be a feasible drug administration route.

## 2. Materials and Methods

### 2.1. Materials

Davisco Foods International (Le Sueur, MN, USA) and Cremer (Hamburg, Germany) kindly donated whey protein isolates BiPro^®^ and Miglyol 812 N^®^, respectively. The whey protein comprised at least 97% of dry basis protein, mainly β-lactoglobulin and α-lactalbumin protein. Miglyol 812 N^®^ was kindly donated by Cremer (Hamburg, Germany). Hyaluronic acid sodium (from *Streptococcus equi*), maize amylopectin, alginic acid sodium, and griseofulvin (from *Penicillium griseofulvin*—97.0–102.0%) were purchased from Sigma-Aldrich. KCl and citric acid anhydrous were also purchased from Sigma-Aldrich. NaH_2_PO_4_ and Na_2_HPO_4_ anhydrous, 1 N HCl, 1 N NaOH, and MeOH were purchased from Sigma-Aldrich (Kempton Park, South Africa). Acetonitrile, LiChrosolv^®^, was acquired from Merck (Kempton Park, South Africa).

### 2.2. Aqueous and Oil Phase Preparation

A suspension containing 2% (*w*/*w*) griseofulvin was freshly prepared by dispersal in medium-chain triglycerides, Miglyol 812 N^®^ by sonication (UP200St ultrasonic bath, Hielscher Ultrasonics, Teltow, Germany), preceding emulsification in water. Whey protein, 4% (*w*/*w*), was hydrated in citrate-phosphate buffer pH 5.0 for ~60 min. Hyaluronic acid, 0.4% (*w*/*w*), alginic acid, 0.4% (*w*/*w*), and amylopectin, 2% (*w*/*w*), were transferred to citrate-phosphate buffer pH 5.0 until completely dissolved. These concentrations were determined as the maximum levels in a pilot dissolution study. 

### 2.3. Emulsion and Dry Emulsion Preparation

The emulsion compositions are shown in Table 1 and the composition of the dry emulsions in Table 2. 

The emulsions were prepared in two steps at 25 °C. Griseofulvin was solubilized in Miglyol 812 N^®^ and then added to the pH 5 buffered water phase comprising 2% (*w*/*w*) weigh protein and sonicated with a UP200Ht handheld ultrasonic homogenizer (Hieschler Ultrasonics GmbH, Teltow, Germany) for 1 min to produce the primary emulsion at a 30% (*w*/*w*) o/w emulsion [19,20].

The secondary emulsion was prepared such that the concentrations of the formulation ingredients indicated in Table 1 and Table 2 were reached. Briefly, the primary emulsion was sonicated in a pH 5 buffered solution containing only 1 of the indicated polysaccharides. The sonication procedure was the same as for the primary emulsions [19,20].

The dried emulsions were prepared by lyophilization. The emulsions were refrigerated for 3 h to reach 4 °C. Subsequently, the emulsions were stored for 12 h at −80 °C before lyophilization. Lyophilization was conducted in a VirTis freeze drier (SP Scientific, Gardiner, NY, USA) for 24 h, as described in a published method [33]. The drying chamber was set at 25 °C and the cooling chamber at −50 °C. A vacuum of 10^−2^ mbar was employed [20,33].

Redispersion of the dried emulsions was conducted in 2 mL deionized water by shaking the appropriate quantity of the lyophilized powder and leaving it for 30 min to equilibrate [20]. 

### 2.4. Quartz Crystal Microbalance Studies

A QCM200 quartz crystal microbalance equipped with a 5 MHz crystal resonator (Stanford Research Systems, Sunnyvale, CA, USA) was employed for all QCM studies. Cr/Au quartz crystals (Standford Research Systems) were employed, which produced a resonating frequency of ~5 MHz when inserted into the crystal resonator. All studies were conducted at an ambient temperature of 25 ± 1 °C. The coating solutions were also maintained at 25 °C. The cumulative mass adsorption was calculated with the Sauerbrey equation, Equation (1) [34], and expressed as µg/cm^2^.

(1) $$\Delta f=-\frac{2f_{0}^{2}}{A\sqrt{\rho_{q}\mu_{q}}}\Delta m$$

where Δ*f* is the change in frequency due to layer sorption; *f*_0_ is the reference frequency of the precisely cut quartz crystal wafers, 5 MHz, in the crystal resonator; Δ*m* is the change in sorbed mass of the thin film; *A* is the effective exposed area of the crystal face, ~0.4 cm^2^; the density of quartz, *ρ_q_*, is ~2.65 g/cm^3^; and the shear modulus of the quartz crystal, *µ_q_*, is ~2.95 g/cm/s^2^. For our QCM system, Equation (1) reduces to the expression Δ*f* = 56.6 Hz/µg/cm^2^.

The thickness of the sorbed layer is estimated from the mass adsorption by knowing the density of the polysaccharides and whey protein, which ranged between 1 and 1.3 g/cm^3^. It should be noted that the thickness could be a function of a ”fuzzy” layer, not a perfectly flat one. Equation (2) is used to estimate the layer thickness:

(2) $$T_{f}=\frac{\Delta m}{\rho_{f}}$$

*T_f_* is the film thickness in cm, Δ*m* is the sorbed mass in µg/cm^2^, and *ρ_f_* is the density of the film material in g/cm^3^.

The QCM impedance analysis method was applied to monitor the sorption interaction between griseofulvin and the polymers and the polymer–polymer interactions. QCM showed the efficiency of the LbL self-assembly technique by measuring the frequency–time profile. The griseofulvin was dissolved in methanol and transferred to a quartz crystal wafer and then dried to constant weight by evaporation. Parasitic capacitance [35], a phantom frequency resonance attributed to dipping in and removal from different coating solutions, was canceled for each coating step to ensure accurate frequency–time profiles. 

Whey protein was adsorbed to the surface of a griseofulvin-coated quartz crystal electrode first and the frequency–time profile was measured. Then, whey protein was coated onto the griseofulvin layer, again followed by measuring the frequency–time profile. Lastly, a layer of the selected polysaccharide was coated onto the whey protein layer. Thus, a single bilayer [36] of whey protein/polysaccharide was coated for all LbL-nanocoated formulations [21]. These coated emulsions were lyophilized to yield dried emulsions. Redispersion of the lyophilized emulsions was investigated to establish the efficiency of biopolymer stabilization.

### 2.5. pH Measurement

Change in pH (Metrohm 914 pH/conductometer, Sandton, South Africa) of the fresh and redispersed emulsions was recorded on days 0, 1, and 7. 

### 2.6. Zeta Potential Measurements 

The zeta potential was determined using a Malvern Zetasizer Nano ZS°2000 (Malvern Instruments, Malvern, UK). Redispersed emulsions were diluted 1:3000 (*v*/*v*) with citrate-phosphate buffer at pH 5.0. At least 13 readings were taken per sample and were performed in triplicate for each of the formulations on days 0, 1, and 7 to determine the zeta potential.

### 2.7. Sample Visualization

Secondary emulsions, lyophilized emulsions, and redispersed emulsions were visualized by light microscopy (Motic, Hong Kong) on the day of preparation. The microscope was equipped with a Moticam 3 camera and Motic Images Plus 2.0 software (Motic, Hong Kong). The samples were colored with a water-based dye to ensure proper contrast between the aqueous and oil phase.

### 2.8. Particle Size Analysis

Light scattering was used to determine particle size and distribution at days 0 and 7 via a Malvern Mastersizer 2000, equipped with a wet cell Hydro 2000 SM dispersion unit (Malvern Instruments, Malvern, UK). The lyophilized emulsions were diluted with deionized water to produce light scattering obscuration values of 10–20%. An average value was determined by diluting 2 fresh samples per formulation and taking two readings per sample.

### 2.9. Encapsulation Efficiency

Samples of each of the emulsions and redispersed lyophilized emulsions in pH 5.0 buffer were centrifuged for 15 min at 10,000 rpm to separate the supernatant from the emulsion particles. The supernatant was filtered using a syringe filter with a pore size of 0.2 µm. An equal aliquot of the filtered supernatant of each formulation was then analyzed by HPLC-UV at 236 nm (Agilent^®^ 1100 model series, Agilent Technologies, Palo Alto, CA, USA) to determine the extractable amount of griseofulvin in each formulation. The following equation, Equation (3), was used to calculate the percentage encapsulation efficiency:

(3) $$\%EE=100\cdot \frac{T-E}{T}$$

where %*EE* is the percentage encapsulation efficiency as % (*w*/*w*), *T* is the total amount of griseofulvin, and *E* is the amount of the extracted active ingredient from the supernatant of centrifuged samples. The HPLC method is described in Section 2.16.

### 2.10. Release Profiles of Griseofulvin from the Formulations 

The drug release profiles of all 9 formulations were tested in total, i.e., redispersed, emulsions, lyophilized emulsion powders, and emulsions for each of the polysaccharides i.e., hyaluronic acid, amylopectin, and alginic acid. The release profile of the formulations was tested, utilizing cellulose nitrate membranes (0.2 μm pore size, Whatman, Dassel, Germany). The nitrocellulose membranes were chosen since they provided a suitable method to evaluate the release profile of griseofulvin from each formulation [37,38]. Franz-type diffusion cells [39] were fitted with the membrane to expose a diffusion area of 1.13 cm^2^. The diffusion cells were transferred to a heated water bath maintained at 37 ± 1 °C for 24 h. The acceptor phase consisted of a degassed phosphate buffer with 10% MeOH at pH 7.4 to increase the solubility of griseofulvin in the acceptor phase. Preceding the study, the nitrocellulose membranes were soaked overnight in the acceptor phase to equilibrate the membranes. The donor compartment was filled with 1 mL of emulsion. Lyophilized emulsions were weighed to contain equivalent amounts of 180 mg griseofulvin, chosen due to the solubility of griseofulvin at pH 5.0, such that the saturation concentration was far below the saturation concentration so as to maintain sink conditions [40]. These powders were placed directly on the membranes and lightly compressed to ensure contact with the membranes. The weighed amounts of the dry emulsions were redispersed in 1 mL of pure citrate-phosphate buffer pH 5.0. Samples were withdrawn at 0.5, 1, 1.5, 2, 3, 4, 6, and 8 h from the acceptor compartment and then refilled with 2 mL of preheated acceptor phase. The acceptor phase was stirred with a magnetic stirrer at 750 rpm. At each time point, the membrane integrity was tested via electric resistance measurements as described in Section 2.14. The drug release profile was thus determined over 8 h. Analysis was performed with HPLC and is described in Section 2.16.

### 2.11. Photostability

A short-term photostability study was conducted. Samples of the formulated emulsions, lyophilized emulsions, solution of griseofulvin in methanol, and lyophilized griseofulvin powder were placed in open containers at approximately 30 cm from the light source, employing a Q-Sun XE-1 test chamber (Avatar Solutions, Centurion, South Africa) equipped with a daylight B filter to irradiate samples between 290 and 320 nm, 25 ± 1 °C. The photostability testing was conducted according to the ICH Q1B guideline option 1, which provides for significant irradiation below 320 nm. Option 1 is a harsher UV exposure method than option 2 of the guideline [41]. Each sample was analyzed by HPLC at the beginning of the experiment, *t*_0_, to determine the amount of griseofulvin present in each sample. Samples were subsequently taken at 1, 3, and 6 h and analyzed by HPLC to determine the amount of intact griseofulvin, which was expressed as a percentage of the original quantity. Photostability determination studies were conducted in triplicate. The HPLC method is described in Section 2.16.

### 2.12. Oil Leakage

The oil leakage from the lyophilized emulsions was determined by transferring a weighed amount of each of the formulations onto filter paper, covering an area of 1.13 cm^2^. A microscope plate was placed carefully on top of the lyophilized formulations. The distance from the edge of the emulsion particle to the edge of the location of the outer edge of oil outside the particle was measured in triplicate [20].

### 2.13. Human Skin Preparation for In Vitro Studies

The Ethics Committee of the North-West University, Potchefstroom, South Africa (Ethics number: NWU-00114-11-A5, 2018; an example of the informed consent form was submitted) approved the use of white female abdominal skin for in vitro dermal and transdermal absorption studies. The skin was obtained from consenting donors that underwent abdominoplasty surgery. The skin was received frozen and thawed at room temperature before preparation. An electric dermatome (Zimmer Inc., Warsaw, IN, USA) was utilized to remove split-thickness skin of 400 μm, which included the stratum corneum, viable epidermis, and the upper dermis. The dermatomed skin was refrozen at −20 °C until use after transferring it to filter paper with the stratum corneum facing upwards and then covered in aluminum foil.

All skin sections were used within six months after preparation, as is the commonly experimentally proven practice for permeation studies. The skin may be stored for up to 1 year at −20 °C to conduct permeation studies [42,43,44]. The dermatomed skin was thawed at room temperature immediately before use and cut into adequate-sized circular pieces to cover the 1.13 cm^2^ area of the diffusion cells that separated the donor and acceptor compartment. 

### 2.14. In Vitro Human Skin Absorption Study

The human skin tissue of 3 different donors was utilized to complete the in vitro dermal and transdermal studies of all nine formulations, with 6 diffusion cells per skin donor. Thus, the average of 18 experiments is presented as the absorption of a particular formulation [19,20,45]. The circularly dermatomed skin was placed on the acceptor compartment of the Franz-type diffusion cells. The electric resistance of the skin was determined by using a Tinsley LCR Databridge Model 6401 (Tinsley Precision Instruments, Croydon, UK) to ascertain the skin integrity [45]. Both the donor and the acceptor phase were filled with 0.9% KCl solution. The cells were then placed in a heated water bath at 37 ± 1 °C and allowed to equilibrate for 30 min. The reading was determined at 1 kHz with a maximum voltage of 300 mV root-mean-square average in the parallel equivalent circuit mode, using an alternating current [46]. Only skin samples with an electric resistance higher than 10 kΩ were considered suitable for the studies [47,48,49]. The compartments of the Franz-type diffusion cells were emptied, and the acceptor compartments were filled with 2 mL of pH 7.4 phosphate buffer containing 10% methanol for increased solubility of griseofulvin, while the donor compartments were filled with either 1 mL of emulsion, equivalent amounts of dry emulsion, 180 mg, or equivalent amounts of the dry emulsion were redispersed in 1 mL of pure citrate-phosphate buffer pH 5.0. The amount of drug ensured that saturation could never be achieved in the medium [40]. The acceptor phase was stirred magnetically at 750 rpm. Next, 0.2 mL acceptor phase was withdrawn after 24 h and analyzed by HPLC. The temperature of the heated water bath was maintained at 37 ± 1 °C for the duration of the study.

### 2.15. In Vitro Skin Absorption Sample Preparation for Analysis by HPLC

The skin samples were removed from the Franz cells after completion of the 24 h study and pinned onto filter paper with the stratum corneum facing upwards. The formulation was gently removed from the surface of the skin sample by dabbing the skin surface with a paper towel. Then, 3 M Scotch^®^ Magic™ tape was used to remove the stratum corneum from the skin samples. The first strip was discarded and was followed by the collection of fifteen strips, placed in 5 mL of methanol per skin sample. The remainder of the skin sample was cut into small pieces and placed in methanol. The samples in the methanol were stored at room temperature for at least 12 h to extract the griseofulvin in the samples. These samples were then mixed and filtered through hydrophilic PVDF syringe filters with a pore size of 0.45 μm (Agela Technologies Inc., Wilmington, DE, USA) and analyzed by HPLC.

### 2.16. HPLC-UV Method

An in-house method was devised. An Agilent^®^ 1100 Series HPLC system (Agilent Technologies, Palo Alto, CA, USA) was used, equipped with a 1311A quaternary pump, G1313A autosampler, and a diode array detector, set at 236 nm. A reversed-phase C18-2 column (150 × 4.6 mm) with 5 μm particle size (Venusil XBP Agela Technologies, Wilmington, DE, USA) was employed. The mobile phase consisted of degassed 50% acetonitrile and 50% Milli-Q^®^ water. The injection volume was set at 25 µL with a flow rate of 1 mL/min at a run time of ~10 min. All the analyses were performed at 25 ± 1 °C ambient and instrument temperature. The method was developed according to the ICH Q2B guideline for the validation of analytical procedures [50]. 

### 2.17. Statistical Calculations

Statistica 14 (TIBCO Software Inc, Palo Alto, CA, USA) was used for all statistical calculations. A multiple distributions fit analysis of data was executed. The data were evaluated for normal and non-normal distribution according to Kolmogorov–Smirnov (K-S) [51] and Anderson–Darling (A-D) tests [52]. In the text, only K-S *p*-values are shown. The supporting information also shows the A-D *p*-values. Studentized 2-tail *t*-tests were used if only 2 data points were evaluated for differences.

## 3. Results

### 3.1. Quartz Crystal Microbalance

**Figure 1:**
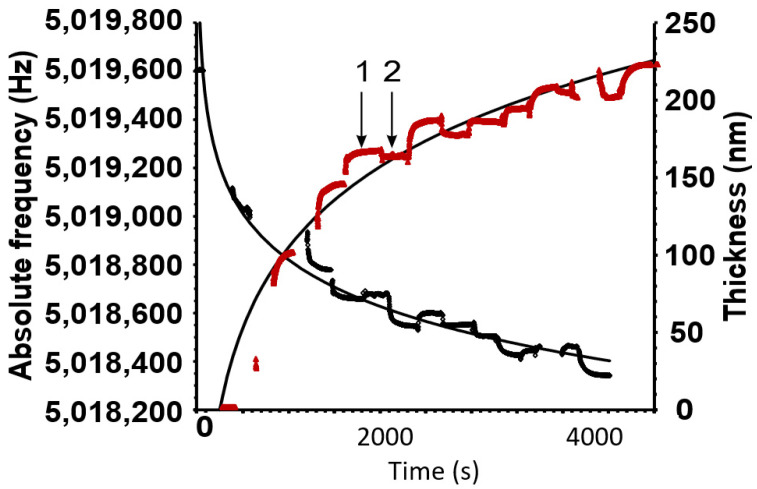
The black curve shows the decrease in frequency as the different polymers are sorbed. The red curve indicates the increase in thickness of the total amount of sorbed polymers. The number 1 represents the excess sorption when the crystal is dipped into a polymer solution. After dipping, the crystal is washed to remove the excess, and at 2, the remaining layer thickness can be found. Conversely, the frequency shows a proportional but negative trend.

As an example of the successive sorption of whey protein and polysaccharide, only a whey protein–amylopectin example is shown in Figure 1. Three bilayers were coated onto the griseofulvin layer.

The sorption of the polymer onto and from the previously absorbed layer reached a maximum and minimum within a short period. The excess polymer was washed off easily and the remaining layer stabilized, as seen at 2. Not shown in the curve are the abrupt fluctuations in the frequency–time profiles of the two curves as the crystal was lifted out of the solution and placed back in. An example of these fluctuations is shown in Figure S1 of the Supplementary Materials.

The example above shows that significant amounts of the polymers could be adsorbed successively. Three bilayers of whey protein and amylopectin could produce a coating of ~225 nm. The trendlines are logarithmic and illustrate that the bonding surface of the crystal and preceding layers is gradually decreasing. However, in free-standing objects, the surface increases concentrically and the coating thickness will, therefore, increase exponentially rather than diminish as for the QCM crystal. The coating thickness for all the single bilayers comprising whey of protein/polysaccharide that were utilized in emulsion formulations produced a film thickness of ~20–30 nm as calculated by Equation (2).

### 3.2. Particle Size Analysis

Table 3 summarizes the particle size measurements of the emulsions and lyophilized emulsions, stabilized with the polysaccharides. The particle size measurements are presented as volume-weighted means (D[4,3]) and surface-weighted means (D[3,2]). It can be seen in Table 3 that the lyophilized emulsions yielded larger particle sizes in comparison to the emulsions, which indicated successful coating. It can be seen that all of the emulsions had D[4,3] sizes ≤ 7 µm on day 0. Furthermore, only the alginic acid-stabilized emulsion showed a significant increase in droplet size (from 7 µm on day 0 to 11 µm on day 7). The lyophilized emulsions indicated D[4,3] of 35–63 µm, as shown in Table 3. It is seen that the hyaluronic acid lyophilized emulsions almost double in size over the 7 days (35 µm on day 0 compared to 66 µm on day 7). On days 0, 1, and 7, microscopy images were taken of the emulsions, redispersed emulsions, and lyophilized emulsion powder. The images represented in Figure 1 are the images taken on day 7. The emulsions and the redispersed emulsions were diluted with buffer to improve the visibility of the droplets. Small droplets were also present in the redispersed lyophilized emulsions.

### 3.3. Sample Visualization 

**Figure 2:**
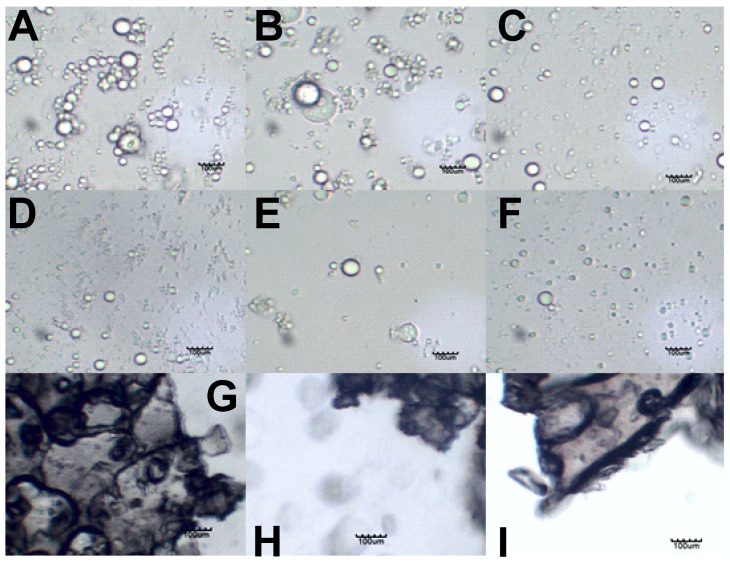
Light microscopy images of emulsions stabilized by (**A**) hyaluronic acid, (**B**) amylopectin, and (**C**) alginic acid on day 7. Redispersed emulsions stabilized by (**D**) hyaluronic acid, (**E**) amylopectin, and (**F**) alginic acid. Lyophilized emulsion powders stabilized by (**G**) hyaluronic acid, (**H**) amylopectin, and (**I**) alginic acid. The scale bar in each image represents 100 µm.

Figure 2 shows the light microscopy images that were produced from the griseofulvin formulations.

Figure 2A–C show the secondary coarse emulsions that were formed by sonication. The alginic acid formulations seemingly produced fewer droplets, which points again to the poor oil encapsulation of alginic acid. Hyaluronic acid and amylopectin produced many more droplets, and these seemed to form clusters of individual droplets, although they did not appear to have coalesced.

Figure 2D–F show that the lyophilized emulsions could be redispersed to form the individual oil droplets of the formulations.

Figure 2G,H show that the dried particles made contact, but the dark LbL nanocoating boundaries around each particle were still present and intact. This explains why individual droplets formed during the redispersion of these particles.

### 3.4. Stability of Griseofulvin in the Formulations

Table 4 summarizes the stability (recovery of intact drug) of griseofulvin in the formulations and the pH measurements of the emulsions over the 7-day test period. In Table 4, the stability of griseofulvin of the preparation is indicated as a percentage recovery after day 7 compared to the availability of griseofulvin in the formulations on day 0—the day of formulation preparation. All the formulations indicated that the stability was higher than 91%. The lyophilized hyaluronic acid emulsions had the lowest stability, with 92% recovery, and the conventional hyaluronic acid emulsions had the highest stability, with virtually no deterioration over 7 days. Furthermore, it was seen that the griseofulvin powder suspended in the oil phase and dissolved in methanol had significantly lower stability values. The pH of the emulsions had no significant change over the 7 days.

### 3.5. Zeta Potential

Table 5 indicates the changes in zeta potential over the 7 days. An increase in zeta potential could be observed from day 0 to 7 for all the formulations except the alginic acid lyophilized emulsion, which showed a slight decrease. No significant changes were observed for the formulations, except for amylopectin emulsions, with an absolute increase of ~10 mV when tested on day 7.

The redispersed emulsions underwent a smaller change in zeta potential over 7 days. It can be speculated that the removal of water from the emulsions to form the powders could result in accelerated relaxation and interdiffusion of the surface bilayer.

### 3.6. Oil Leakage and Encapsulation Efficiency

Table 6 indicates the encapsulation efficiency of emulsions and lyophilized emulsion powders and the oil leakage from the lyophilized emulsion powders on days 1 and 7.

The encapsulation efficiency data indicate high encapsulation efficiency for all the formulations, exceeding 90%, except for the alginic acid stabilized emulsion, with an encapsulation efficiency of ~81%. The oil leakage from the lyophilized emulsion powders is presented as the distance from the lyophilized formulation powder to the oil stain on the paper. It was observed that the stabilized hyaluronic acid had the highest initial oil leakage of 37.3 ± 1.9 mm on day 1. However, after 7 days, the lyophilized emulsion powders stabilized with the use of alginic acid had the highest oil leakage of 68.7 ± 1.2 mm. The lyophilized emulsion powders stabilized with the use of amylopectin had the lowest oil leakage after 7 days, namely 36.7 ± 1.7 mm.

### 3.7. Photostability

**Figure 3:**
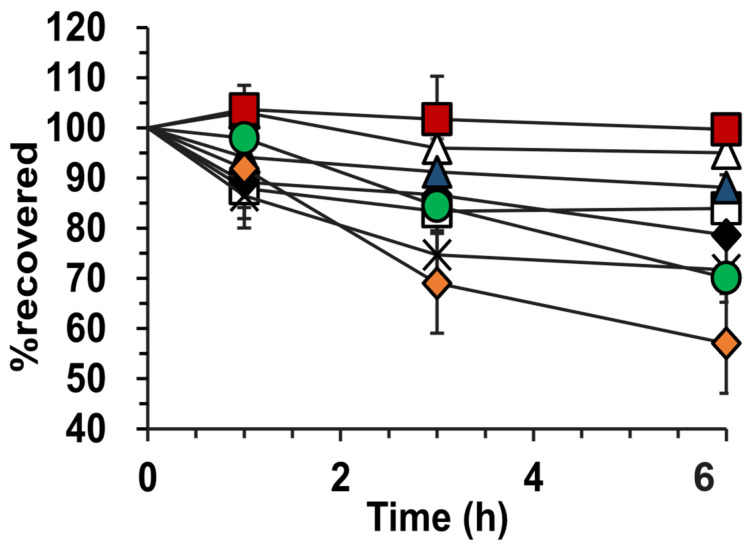
Photostability of griseofulvin-containing formulations. (∆) Hyaluronic-stabilized emulsion, (□) amylopectin-stabilized emulsion, (♦) alginic acid-stabilized emulsion, (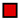) hyaluronic-stabilized lyophilized emulsion, (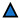) amylopectin-stabilized lyophilized emulsion, (×) alginic acid-stabilized lyophilized emulsion, (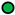) griseofulvin solutions in methanol, and (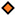) griseofulvin powder. Triplicate experiments were performed.

In Figure 3, the photostability of griseofulvin is indicated as a percentage recovered at 1, 3, and 6 h compared to *t_0_*. The hyaluronic acid-stabilized lyophilized emulsion is shown to have the highest photostability of 99.7 ± 2.3 % and the pure griseofulvin powder had the lowest photostability of 57 ± 1.5%. After comparison of the 6 h data points of the lyophilized hyaluronic acid and pure griseofulvin with a two-tailed Student *t*-test, the difference is significant at *p* = 1.13 × 10^−5^. It can further be noted that the alginic acid-lyophilized emulsion had the lowest photostability of the prepared emulsions at 71.7 ± 3.2%.

### 3.8. Release of Griseofulvin from the Formulations

The release pattern of all formulations followed a non-normal data distribution. The analyses are provided in Figure S2 (Supplementary Materials). The release profiles were evaluated by Kolmogorov–Smirnov tests (K-S tests) and confirmed with Anderson–Darling tests to first determine the type of data distribution and secondly to overlay these distributions and determine if any significant differences were present between curves.

**Figure 4:**
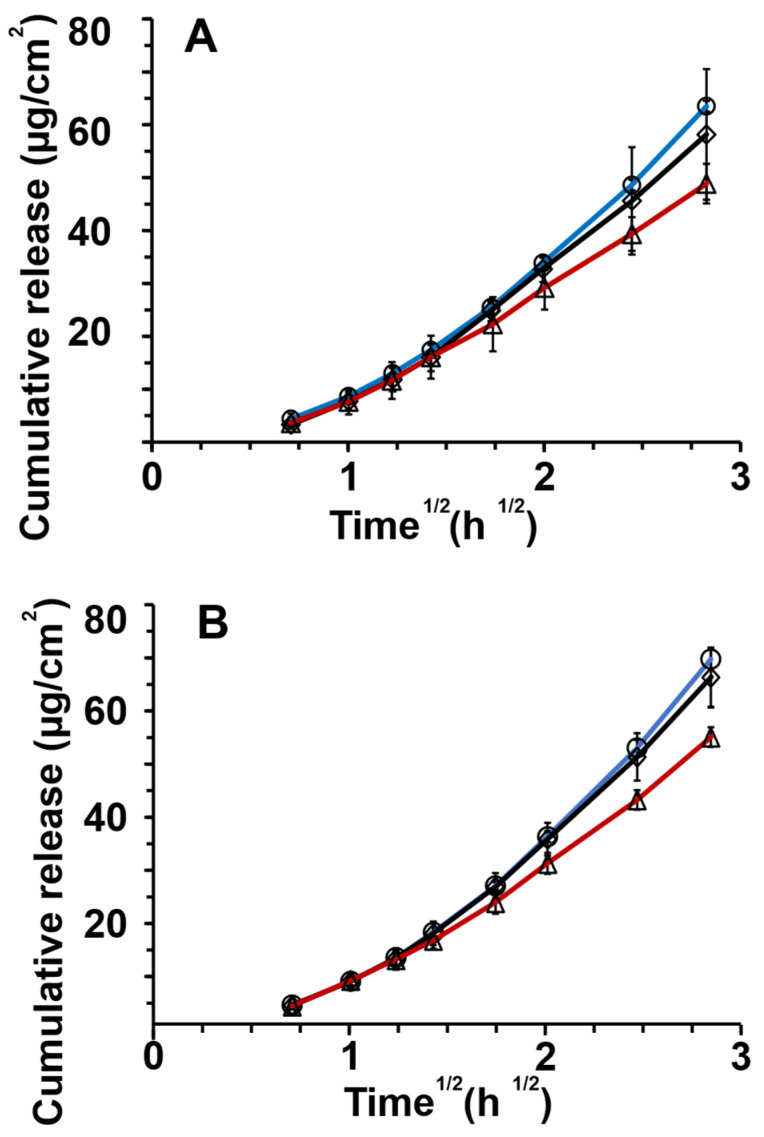

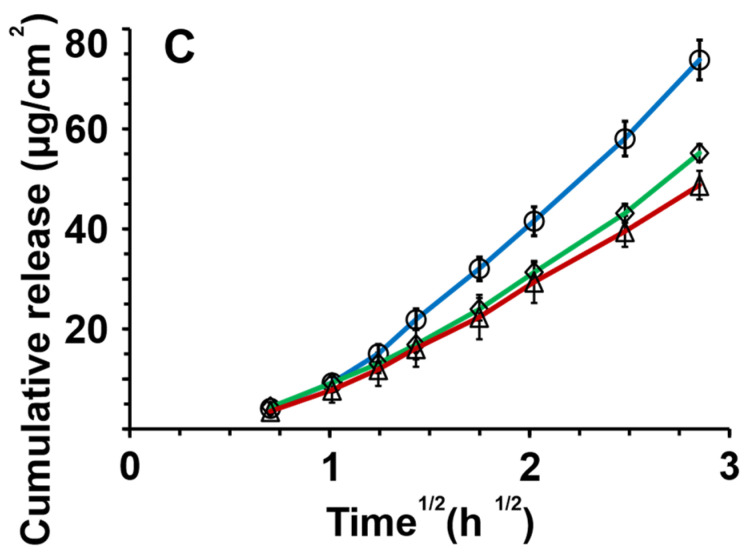
In vitro release of griseofulvin through nitrocellulose membranes for (**A**) hyaluronic acid, (**B**) amylopectin, and (**C**) alginic acid for different formulations: ◊ dry emulsion powders, ◯ redispersed dry emulsions, and △ emulsions. The release data and standard deviations are presented as a linear fit against the square root of time according to the simplified Higuchi equation (*n* = 4).

In Figure 4A, the release profiles from hyaluronic acid-stabilized formulations are shown. The redispersed emulsion fitted a Johnson distribution (K-S *p* > 0.999), lyophilized powders a Gaussian mixture distribution (K-S *p* > 0.999), and emulsions a Gaussian mixture distribution (K-S *p* >0.999). As shown in Figure S3 (Supplementary Materials), the overlap of these distributions was significant formulations; therefore, the release profiles differed insignificantly.

Figure 4B shows the release profiles of amylopectin formulations. Non-normal distributions of the data were observed as seen in Figure S4 (Supplementary Materials). Redispersed emulsions and lyophilized powder both fitted a Johnson distribution (K-S *p* > 0.999). Emulsion profiles fitted a Gaussian mixture distribution (K-S *p* > 0.999). The significant overlap of the distribution-fitted formulation release profiles was found, and the release profiles from amylopectin formulations differed insignificantly.

Figure 4C depicts the release profiles of alginic acid formulations. Redispersed emulsions and lyophilized powders showed Johnson data distribution (K-S *p* > 0.999) as shown in Figure S5 (Supplementary Materials). The emulsion release data showed Gaussian mixture distribution (K-S *p* > 0.999). There was an insignificant difference in the release profiles of the alginic acid formulations. In Tables S1–S6 (Supplementary Materials), the results of the distribution analyses can be seen. Figure S6 (Supplementary Materials) displays all the release curves for all formulations together.

### 3.9. In Vitro Skin Absorption Study

Although a long-term stability study was not performed, it can be envisioned that the various lyophilized polysaccharide formulations will provide better long-term stability, and reconstitution of the lyophilized powders could be performed before topical skin application.

**Figure 5:**
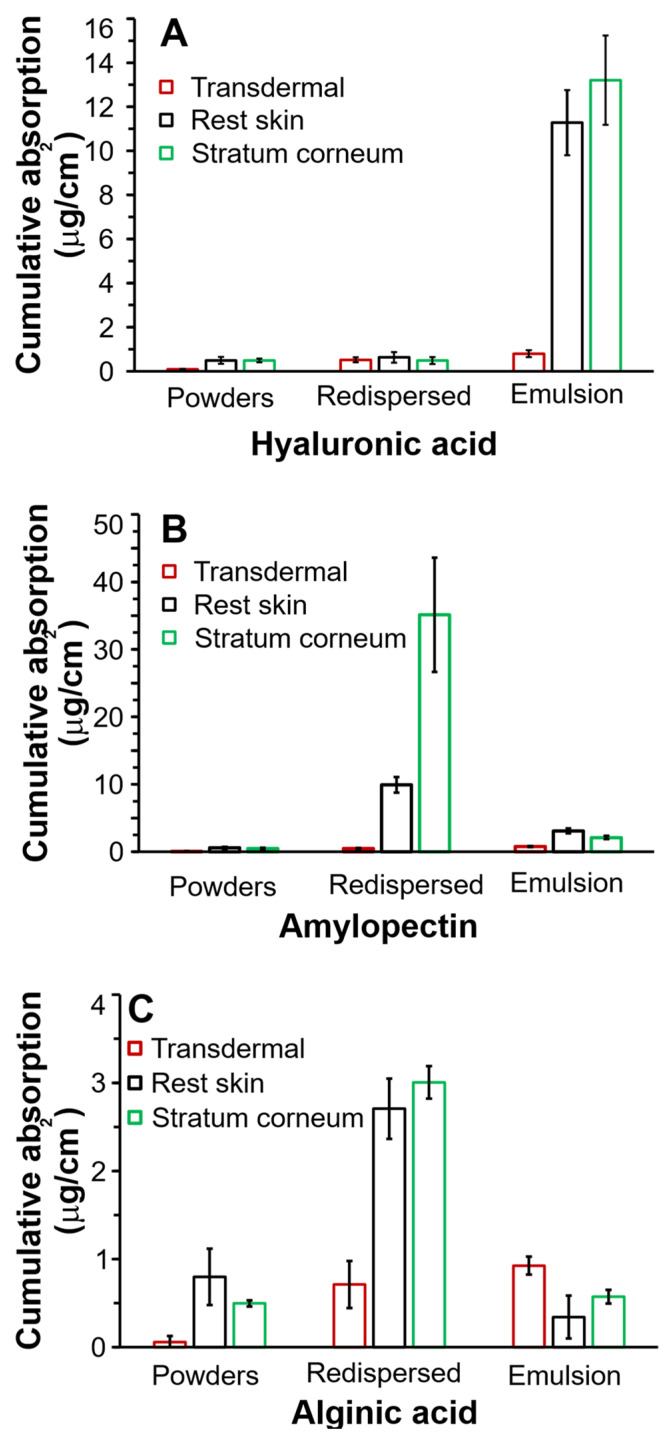
Average in vitro skin absorption data of griseofulvin from (**A**) hyaluronic acid, (**B**) amylopectin, and (**C**) alginic acid-stabilized formulations through human abdominal skin expressed as total amount delivered over 24 h. Three skin donors were used, with 6 repetitions per skin donor per formulation.

Figure 5 indicates the in vitro skin absorption through human abdominal skin of griseofulvin formulations, indicating the effect that different formulations have on the total 24 h absorption of griseofulvin formulations stabilized by different polysaccharides.

It can be seen in Figure 5A that the emulsions stabilized with hyaluronic acid produced the highest absorption (*p* = 0.007), in the stratum corneum, reaching ~13 µg/cm^2^ and ~12 µg/cm^2^ in the rest skin in the 24 h absorbance study compared to the absorption from redispersed and lyophilized powders. Figure 5A–C show that hyaluronic acid-stabilized emulsion will be the most effective formulation regarding drug accumulation from the epidermal to the stratum corneum layers. The lyophilized powder and redispersed emulsion showed minimal, insignificant absorption lower than ~1 µg/cm^2^ in all layers, regardless of the chosen polysaccharide. Insignificant transdermal accumulation, ~1 µg/cm^2^, was also observed for all formulations.

The redispersed emulsions were stabilized with amylopectin, as shown in Figure 5B, and were best targeted by redispersed emulsions compared to the rest skin. The amylopectin powders and emulsions produced ineffective absorption compared to the redispersed emulsions (*p* ≥ 0.26).

Alginic acid formulation absorption, as seen in Figure 5C, showed the lowest griseofulvin absorption by all the skin layers. Although the alginic acid-coated redispersed emulsions were the most effective delivery systems, their stratum corneum accumulation was ~12-fold lower than for redispersed amylopectin emulsions and ~4-fold lower than that of hyaluronic emulsions. The alginic acid formulations were excluded from further statistical analysis.

After comparison of the three polysaccharide formulations regarding the ideal stratum corneum layer absorption, the amylopectin redispersed formulation proved far superior to any other formulation. The hyaluronic dry powders and redispersed emulsion showed insignificant differences in absorption for all skin layers (*p* = 0.25).

## 4. Discussion

### 4.1. Particle Size Analysis, Sample Visualization, Zeta Potential Measurements, Drug Stability, and pH Measurement

The preparation of the emulsions was based on the LbL technique by layering emulsion droplets with a primary layer of whey protein, followed by a second layer of either hyaluronic acid, amylopectin, or alginic acid [53]. After 7 days, the recovery of griseofulvin indicated that all the formulations still contained at least 91% griseofulvin. Approximately 66% and 68% of the griseofulvin was recovered from the griseofulvin oil suspension and methanol solution, respectively. The stabilization of emulsions with polysaccharides increased the stability of griseofulvin. The lyophilized emulsions also showed high retention of the intact drug compared to the solution and suspension. It has been reported that substances encapsulated by biopolymers could be protected from oxidation, chemical, or enzymatic degradation [25,54]. It was found that encapsulating olive oil droplets in emulsions reduced the oxidation of the oil [33]. It was also reported that spray-dried tuna oil that was stabilized with chitosan and lecithin had improved stability against oxidation compared to unprotected oil [15]. This confirms the protective function of the biopolymer layers toward encapsulated griseofulvin. It also advocates for the removal of the aqueous phase to prevent hydrolysis and oxidation of the drug and the oil phase of emulsions. No clear changes were observed in the pH measurements of the emulsions.

The particle size stability was determined over only 7 days. Except for alginic acid-stabilized emulsions and hyaluronic acid-stabilized lyophilized emulsions, which showed an almost doubling in particle size, the formulations showed good stability towards particle size stability. When compared, the lyophilized emulsion formulations indicated larger particle sizes in comparison with the correlating emulsions. The possibility exists to decrease the lyophilized emulsion particle size by spray-drying the emulsions instead of the lyophilization method [55,56]. When comparing the droplet size measurements to the microscopy photographs of the lyophilized emulsions, smaller droplets were observed on the images.

The zeta potential of the emulsions and lyophilized emulsions was shown to be stable over the 7 days by showing < 7% changes in the zeta potential. Exceptions were the hyaluronic acid- and amylopectin-stabilized emulsions, which decreased from −60 mV to −50 mV or by ~18% and from −61 mV to −49 mV or by ~20%, respectively. It is known that structural relaxation and polyelectrolyte interdiffusion could affect the net surface excess of a particular species [57]. The hyaluronic and amylopectin emulsions showed a decrease in the zeta potential magnitude. This could be attributed to the interdiffusion of the polyelectrolytes over the 7 days. A future study should investigate this further.

The stability of the griseofulvin in the formulations over 7 days was compared to the amount of active ingredient present on day 0 of preparation and after the 7 days in each formulation. It was observed that all the formulations had significantly higher stability when compared to a suspension made of griseofulvin in Miglyol 812 N^®^ and a solution of griseofulvin in methanol, indicating the ability of layers of polysaccharides and the drying of emulsions increasing the stability of the active ingredient. No significant differences were observed between the emulsions and lyophilized emulsion powders when comparing the stability of the active ingredient.

### 4.2. Encapsulation Efficiency and Oil Leakage

The encapsulation data for all the formulations indicate the high encapsulation capabilities of the polysaccharides. Emulsions stabilized by alginic acid displayed significantly lower encapsulation efficiency, indicating that a higher concentration of alginate may be needed, or encapsulating the oil phase with more than one bilayer of protein/alginate with the use of the LbL technique.

The leakage of oil from the lyophilized powder formulations could result in instabilities and is also an indication of the adequate encapsulation of the oil phase. By oil leaking from formulations, griseofulvin in the oil phase is then exposed to the environment, resulting in reduced stability and bioavailability. Oxidation of the oil phase could also result due to the leakage from the formulations. The greater the distance from the formulations to the oil stain barrier, the greater the oil leakage from the formulation. The lyophilized emulsion powders stabilized with amylopectin had the lowest oil leakage on days 1 and 7, indicating efficient encapsulation of the oil phase containing the griseofulvin. The lyophilized emulsion powders, stabilized with hyaluronic acid, had the highest initial oil leakage on day 1. However, after 7 days, no significant change was observed when compared to day 1. This suggested that the encapsulated oil effectively prevented leakage of the drug. The stability data from the lyophilized emulsion powders, shown in Table 4, could also indicate that the hyaluronic acid-stabilized lyophilized emulsion powders had the lowest stability on day 7, with a high initial loss of the active ingredient in the non-encapsulated oil droplets. The formulations coated with amylopectin showed high encapsulation efficiency, resulting in low oil leakage and sufficient stability of the griseofulvin.

The lyophilized emulsion powders stabilized with alginic acid showed the highest oil leakage. These results correlate with the data obtained for the encapsulation efficiency. All the alginic acid-coated formulations showed the lowest encapsulation efficiency compared to any of the other formulations. Conversely, high active ingredient stability was found for alginic acid coatings. This could be due to a large initial loss of non-encapsulated griseofulvin during the preparation of the emulsion, followed by the effective protection of the low amount of encapsulated drug during the 7 days.

### 4.3. Photostability

The photostability studies indicated the improved stability of griseofulvin towards photodegradation in emulsions nanocoated with hyaluronic acid and amylopectin. These results correlate with results obtained for entrapped amlodipine, which also showed improved photostability compared to untreated amlodipine [8]. The lyophilized emulsions stabilized with amylopectin had the highest photostability, followed by the hyaluronic-stabilized emulsions. Amylopectin- and hyaluronic acid-stabilized emulsions differed by less than 5% regarding their initial photostability measurement. All the emulsions and lyophilized powders stabilized with hyaluronic acid and amylopectin indicated a photostability of higher than 80%.

Both the griseofulvin powder and solution indicated a rapid decrease in photostability after 1 h, with the highest photodegradation of up to 43% compared to the initial measurement. The preparations stabilized with alginic acid indicated no significant improvements in photostability when compared to the griseofulvin powder and solution. When compared to encapsulation efficiency results and oil leakage, alginic acid formulations showed the lowest encapsulation efficiency and highest oil leakage, significantly reducing the photostability of the drug due to higher quantities of the drug exposed to the UV-B light source.

### 4.4. Drug Release Profiles

When comparing the data obtained from the release profile of griseofulvin through cellulose nitrate membranes, shown in Figure 4, it can be observed that neither the type of formulation nor whey protein/polysaccharide had significant effects on the release pattern of griseofulvin. This indicates that the delivery system does not hamper drug release. However, the %EE of alginic acid emulsions suggested excluding this formulation. The magnitude of electrostatic interaction between the oil phase, griseofulvin, and polysaccharides could also have influenced the data. The zeta potential values of all stability samples at day 7 were all around −50 mV. The electrostatic interactions could be investigated in the future—in this study, zeta potential did not seem to influence release. The polysaccharide layer thickness could also play an important role in the release of griseofulvin; however, poor %EE with alginic acid seemingly produced lower rates and extents of release.

### 4.5. In Vitro Skin Absorption

It was shown that the different polysaccharides and types of formulations influenced the area in which the drug localized and accumulated in the in vitro human skin absorption studies. The electric charge of the skin and formulation could have affected the skin, but all the formulations had a zeta potential at day 7 of around −50 mV. This suggested that the zeta potential magnitude of the formulations did not significantly influence the in vitro absorption of the griseofulvin into or through human skin from the formulations.

The in vitro human skin absorption study indicated that griseofulvin accumulated in the stratum corneum on the surface of the human skin model. The accumulation of griseofulvin has also been observed following oral administration within a period of 8 h. The oral bioavailability varied between 31 and 63% after administration of a 500 mg dose with a blood plasma level reaching only ~3 µg/mL. Following this, a maximum level of approximately 450 ng/mL was attained in the stratum corneum, which is still considered therapeutic [58,59]. Our best amylopectin LbL delivery system significantly exceeded this accumulation by achieving ~35 mg/mL in the same period of 24 h. This observation holds the significant promise that topical LbL-coated formulations could provide a significantly more successful treatment of epidermal fungal infection compared to systemic treatment. In this regard, redispersed amylopectin powders resulted in the highest deposition of the drug in the stratum corneum, reaching ~35 µg/cm^2^ griseofulvin. The second most effective formulation to deliver griseofulvin to the stratum corneum was the hyaluronic acid-containing emulsions, which attained ~13 µg/cm^2^, which was ~2.7-fold less than the amylopectin lyophilized powder. The alginic acid powders were the best formulations for stratum corneum delivery; however, they showed only ~3 µg/cm^2^, which was ~12-fold lower than the redispersed amylopectin emulsions. Under the experimental conditions of this study, we advise against using alginic acid to produce an effective topical delivery system.

## 5. Conclusions

This study illustrated that (i) LbL nanocoating successfully produced stable emulsions and lyophilized emulsions by (ii) employing whey protein as the initial layer followed by a layer of a polysaccharide of either hyaluronic acid, alginic acid, or amylopectin to (iii) produce efficient topical delivery systems for topical fungal infections. The successful application of whey protein and polysaccharides proved that LbL nanocoatings with environmentally benign materials are possible.

Employing amylopectin, which is poorly soluble in the aqueous phase, a Pickering emulsion was formulated. These emulsions were also successfully formulated into lyophilized emulsions. The emulsions and the lyophilized emulsions showed good griseofulvin stability when compared to an oil suspension and a methanol solution of griseofulvin. Particle sizing also proved to be stable, except for alginic acid emulsions and hyaluronic acid lyophilized emulsions. Photostability evaluation indicated the enhanced stability of the emulsions and the lyophilized emulsions containing hyaluronic acid and amylopectin when compared to the griseofulvin solution and powder. The release profiles and in vitro human skin absorption profiles of the griseofulvin formulations suggest that reconstitution before topical application will ensure successful treatment.

We conclude that a single LbL bilayer formulation of amylopectin or hyaluronic acid in conjunction with whey protein produced a feasible topical delivery system for griseofulvin to treat skin fungal infections.

## Figures and Tables

**Table 1:** Composition of secondary emulsions. Whey protein formed the first layer in all cases followed by the polysaccharide. Compositions are provided as % (*w*/*w*).

Ingredient	Hyaluronic Acid	Amylopectin	Alginic Acid
Griseofulvin	0.3	0.3	0.3
Miglyol 812 N^®^	14.7	14.7	14.7
Whey protein	2.0	2.0	2.0
Hyaluronic acid	0.2	-	-
Amylopectin	-	1.0	-
Alginic acid	-	-	0.2
Water	82.8	82.0	82.8

**Table 2:** Composition of dry emulsions as % (*w*/*w*). Whey protein formed the first layer in all cases followed by the polysaccharide.

Ingredient	Hyaluronic Acid	Amylopectin	Alginic Acid
Griseofulvin	1.58	1.58	1.58
Miglyol 812 N^®^	86.20	81.75	86.20
Whey protein	11.11	11.11	11.11
Hyaluronic acid	1.11	-	-
Amylopectin	-	5.56	-
Alginic acid	-	-	1.11

**Table 3:** Particle sizing of the griseofulvin formulations.

		Day 0			Day 7		
Formulation	Polysaccharide	D[4,3] (µm)	D[3,2] (µm)	Span	D[4,3] (µm)	D[3,2] (µm)	Span
Emulsions	Hyaluronic acid	5.18 ± 1.1	2.86 ± 0.1	1.418 ± 0.01	6.801 ± 0.3	3.282 ± 0.04	1.703 ± 0.05
	Amylopectin	6.48 ± 0.6	2.63 ± 0.01	1.962 ± 0.04	8.924 ± 0.4	3.232 ± 0.01	2.400 ± 0.07
	Alginic acid	6.98 ± 0.7	3.59 ± 0.02	1.515 ± 0.02	11.13 ± 1.0	3.830 ± 0.01	1.962 ± 0.02
Redispersed lyophilized emulsions	Hyaluronic acid	34.9 ± 14.2	3.12 ± 0.3	11.59 ± 0.7	66.36 ± 1.3	4.818 ± 0.07	3.324 ± 0.07
	Amylopectin	63.0 ± 15.8	4.89 ± 0.7	6.616 ± 1.1	71.99 ± 2.5	6.166 ± 0.1	3.294 ± 0.2
	Alginic acid	50.8 ± 3.8	3.30 ± 0.1	15.12 ± 3.4	54.86 ± 2.7	3.412 ± 0.1	7.974 ± 0.5

**Table 4:** Stability (%recovery) of griseofulvin and pH measurements of emulsions and lyophilized emulsions ± standard deviation.

		%Recovery	pH	
Formulation	Polysaccharide	Day 7	Day 0	Day 7
Emulsions	Hyaluronic acid	100.6 ± 3.2	5.1	4.9
	Amylopectin	98.5 ± 1.8	5.1	4.9
	Alginic acid	98.7 ± 1.7	5.1	4.9
Lyophilized powder	Hyaluronic acid	91.8 ± 0.8		
	Amylopectin	94.0 ± 0.8		
	Alginic acid	98.0 ± 0.8		
Oil suspension		66.4 ± 2.2		
Methanol solution		67.9 ± 0.5		

**Table 5:** Zeta potential measurements of emulsions and redispersed lyophilized emulsions and the oil leakage from lyophilized emulsion powders ± S.D.

		Zeta Potential (mV)	
Formulation	Polysaccharide	Day 0	Day 7
Emulsions	Hyaluronic acid	−59.9 ± 1.5	−49.3 ± 0.1
	Amylopectin	−61.0 ± 0.8	−49.0 ± 2.1
	Alginic acid	−54.7 ± 2.4	−50.6 ± 0.4
Redispersed lyophilized emulsion	Hyaluronic acid	−54.1 ± 1.7	−52.7 ± 2.8
	Amylopectin	−55.1 ± 1.5	−52.3 ± 0.8
	Alginic acid	−51.8 ± 0.5	−53.9 ± 0.7

**Table 6:** Encapsulation efficiency (%) of emulsions and lyophilized emulsion powders and oil leakage from lyophilized emulsions powders on day 1 and day 7 ± standard deviation.

		Encapsulation Efficiency (%EE)	Oil Leakage (mm)	
Formulation	Polysaccharide	%	Day 1	Day 7
Emulsion	Hyaluronic acid	97.8 ± 0.5		
	Amylopectin	94.7 ± 0.2		
	Alginic acid	81.0 ± 1.6		
Lyophilized emulsion powder	Hyaluronic acid	96.3 ± 0.3	37.3 ± 1.9	42.3 ± 2.1
	Amylopectin	96.0 ± 0.5	12.0 ± 0.8	36.7 ± 1.7
	Alginic acid	94.3 ± 0.5	21.7 ± 1.7	68.7 ± 1.2

## Supplementary Materials

The following supporting information can be downloaded at: https://www.mdpi.com/article/10.3390/pharmaceutics14030554/s1, Figure S1: A complete frequency–time profile of a quartz crystal coating process; Figure S2: Release profiles of griseofulvin through cellulose nitrate membranes for (A) redispersed emulsions, (B) dry powders, and (C) emulsions. Effect of polymers: △ hyaluronic acid, ◊ amylopectin, and ◯ alginic acid. The release data are presented as a fit for the simplified Higuchi equation (*n* = 4); Figure S3: Overlay of release data points of hyaluronic acid formulations according to a quantile–quantile plot; Figure S4: Overlay of release data points of amylopectin acid formulations according to a quantile–quantile plot; Figure S5: Overlay of release data points of alginic acid formulations according to a quantile–quantile plot; Figure S6: All cumulative release curves of griseofulvin from all the investigated formulations; Table S1: Non-parametric data distribution fits—release profiles of redispersed emulsions; Table S2: Non-parametric data distribution fits—release profiles of lyophilized powders; Table S3: Non-parametric data distribution fits—release profiles of emulsions; Table S4: Non-parametric data distribution fits—release profiles of hyaluronic acid formulations; Table S5: Non-parametric data distribution fits—release profiles of amylopectin formulations. Table S6: Non-parametric data distribution fits—release profiles of alginic acid formulations.
