# Supplementary material for: In Vitro Skin Delivery of Griseofulvin by Layer-by-Layer Nanocoated Emulsions Stabilized by Whey Protein and Polysaccharides
Source: Pharmaceutics. 2022 Mar 2;14(3):554. doi: 10.3390/pharmaceutics14030554 (PMC8949154; doi:10.3390/pharmaceutics14030554)
Supplement: Supplementary file 1 [file pharmaceutics-14-00554-s001.zip › pharmaceutics-1586049-supplementary.pdf]

pharmaceutics

MDPI

Supplementary Materials: In Vitro Skin Delivery of Griseofulvin by Layer-by-Layer Nanocoated Emulsions Stabilized by Whey Protein and Polysaccharides

Daniel P. Otto, Anja Otto and Melgardt M. de Villiers

Supplementary Materials

S1. QCM

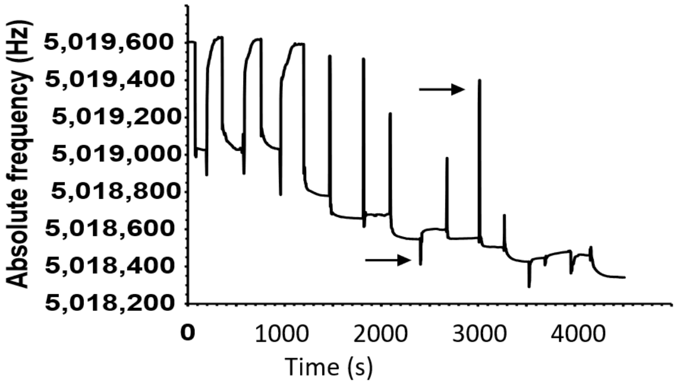

Figure S1. A complete frequency–time profile of a quartz crystal coating process.

In Figure S1, the bottom arrow indicates a sudden drop in frequency as the crystal is dipped into the solution.

As the crystal enters the liquid, significant force is experienced, which only dissipates after time. The top arrow indicates when the crystal is being lifted from the solution. This is experienced as a loss of mass since the liquid drips from the crystal. These sharp fluctuations are artifacts and do not reflect sorption behavior. The parasitic capacitance is a result of the dipping and lifting of the crystal into and out of the liquid and air media.

S2. Release Profiles Classified According to the Formulation or Polysaccharide

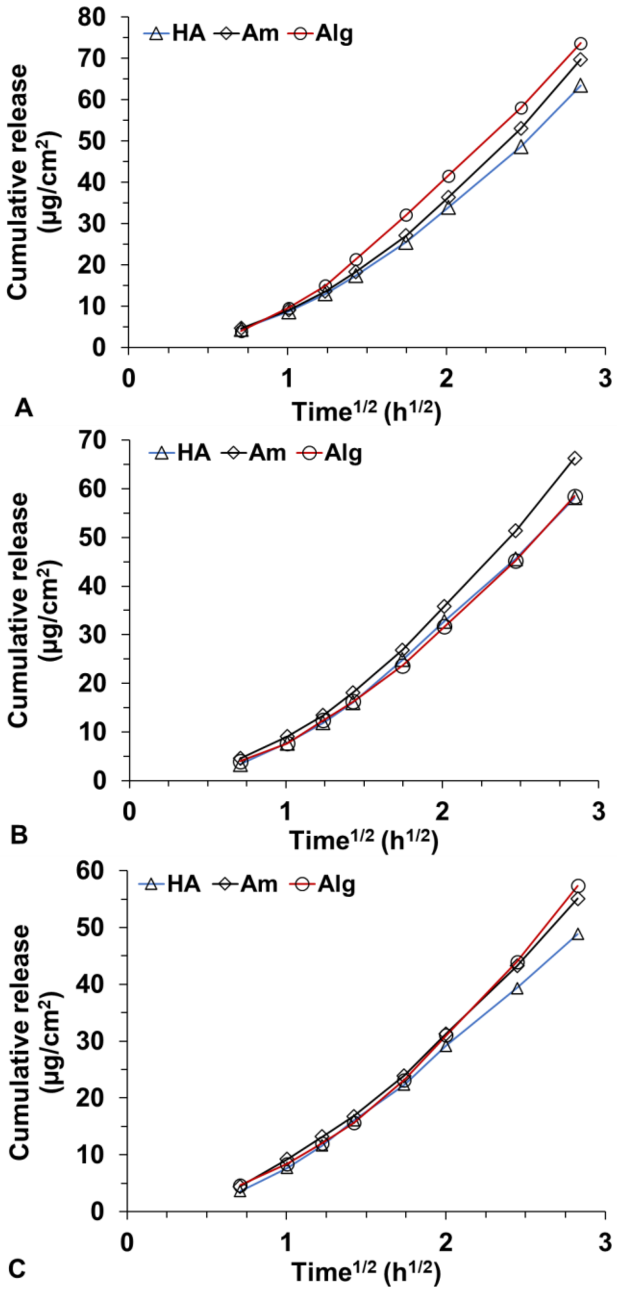

Figure S2. Release profiles of griseofulvin through cellulose nitrate membranes for (**A**) redispersed emulsions, (**B**) dry powders, and (**C**) emulsions. Effect of polymers: △ hyaluronic acid, ◊ amylopectin, and ○ alginic acid. The release data are presented as a fit for the simplified Higuchi equation (n = 4).

In Figure S2, the error bars are not indicated; however, they have been shown in the main text.

Refer to Tables S1–S6 for statistical analysis of all the release data distribution fits.

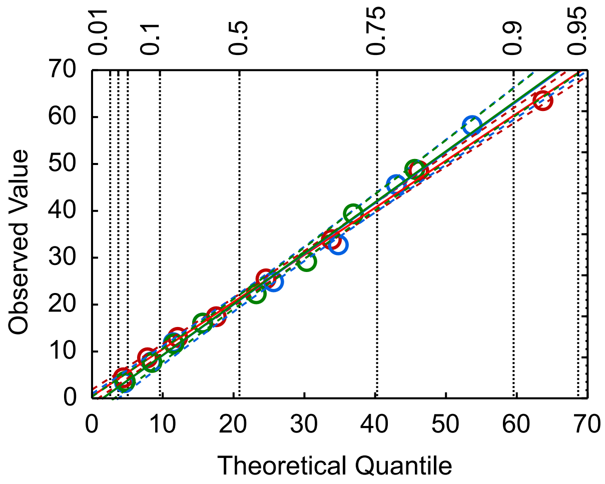

Figure S3. Overlay of release data points of hyaluronic acid formulations according to a quantile–quantile plot.

Figure S3 shows virtually perfect fits of the non-normal data distributions for redispersed emulsions (red), lyophilized powders (blue), and emulsions (green). The confidence intervals overlap significantly and confirm that the release profiles differed insignificantly (K-S $p > 0.999$).

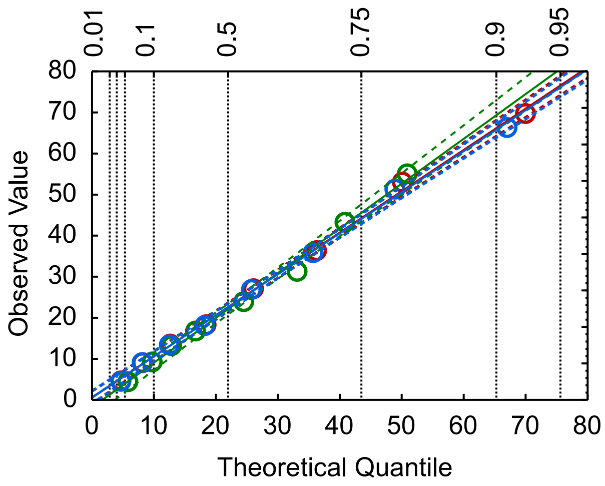

Figure S4. Overlay of release data points of amylopectin acid formulations according to a quantile–quantile plot.

Figure S4 shows virtually perfect fits of the non-normal data distributions for redispersed emulsions (red), lyophilized powders (blue), and emulsions (green). The confidence intervals overlap significantly and confirm that the release profiles differed insignificantly (K-S $p > 0.999$).

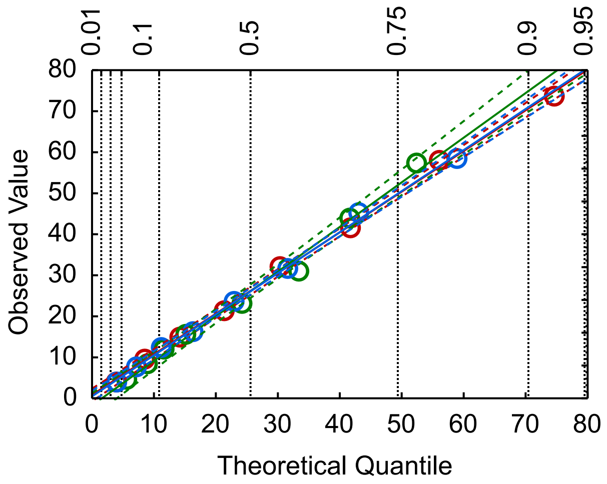

Figure S5. Overlay of release data points of alginic acid formulations according to a quantile–quantile plot.

Figure S5 shows virtually perfect fits of the non-normal data distributions for redispersed emulsions (red), lyophilized powders (blue), and emulsions (green). The confidence intervals overlap significantly and confirm that the release profiles differed insignificantly (K-S $p > 0.999$).

Table S1. Non-parametric data distribution fits—release profiles of redispersed emulsions.

Distribution Rank	K-S d *	K-S *p*-Value	A-D Stat **	A-D *p*-Value
Johnson SB	0.097405	0.999977	0.108921	0.999825
Gaussian Mixture	0.104955	0.999891	0.117417	0.999645
Half Normal	0.108017	0.999810	0.139012	0.999011
Log Normal	0.112250	0.999613	0.135492	0.999131
General Extreme Value	0.117822	0.999094	0.159567	0.997552
Weibull	0.123363	0.998057	0.146307	0.998740
Normal	0.176713	0.928974	0.297098	0.939311
Triangular	0.197994	0.856448	8.868846	0.000051
General Pareto	0.225951	0.730629	4.639397	0.004565
Rayleigh	0.258317	0.574161	0.837735	0.450468

* K-S; Kolmogorov–Smirnov; ** A-D; Anderson–Darling.

Table S2. Non-parametric data distribution fits—release profiles of lyophilized powders.

Distribution Rank	K-S d *	K-S *p*-Value	A-D Stat **	A-D *p*-Value
Gaussian Mixture	0.098022	0.999974	0.115135	0.999697
Johnson SB	0.103379	0.999920	0.112785	0.999748
Half Normal	0.110386	0.999715	0.128739	0.999344
Weibull	0.119299	0.998882	0.149016	0.998632
General Extreme Value	0.124202	0.997831	0.174705	0.995400
Folded Normal	0.126600	0.997137	0.136526	0.999097
Log Normal	0.133588	0.994361	0.168442	0.996366
Normal	0.179490	0.921006	0.266711	0.960758
General Pareto	0.191592	0.880882	8.837626	0.000053
Rayleigh	0.224054	0.739831	4.567513	0.004945

* K-S; Kolmogorov–Smirnov; ** A-D; Anderson–Darling.

Table S3. Non-parametric data distribution fits—release profiles of emulsions.

Distribution Rank	K-S d *	K-S *p*-Value	A-D Stat **	A-D *p*-Value
Gaussian Mixture	0.093326	0.999992	0.112031	0.999763
Johnson SB	0.095535	0.999985	0.108815	0.999827
Half Normal	0.107780	0.999818	0.153895	0.998198
Weibull	0.116572	0.999246	0.142620	0.998880
General Extreme Value	0.122360	0.998298	0.166359	0.996664
Log Normal	0.125671	0.997411	0.172000	0.995830
Normal	0.154133	0.975730	0.222080	0.983135
General Pareto	0.171512	0.942577	4.384606	0.006026
Triangular	0.200831	0.844979	8.796511	0.000055
Rayleigh	0.203000	0.835967	0.556686	0.684399

* K-S; Kolmogorov–Smirnov; ** A–D; Anderson–Darling.

Table S4. Non-parametric data distribution fits—release profiles of hyaluronic acid formulations.

		K-S *p*-Value	
Distribution	Redispersed	Dried	Emulsions
Gaussian Mixture	0.999891	0.999917	0.999984
Johnson SB	0.999977	0.999940	0.999826
Half Normal	0.999810	0.999711	0.998795
Weibull	0.998057	0.997524	0.997543
General Extreme Value	0.999094	0.998568	0.998693
Log Normal	0.999613	0.999400	0.999652
Normal	0.928974	0.923058	0.939447
General Pareto	0.730629	0.726308	0.868306
Triangular	0.856448	0.862547	0.888420
Rayleigh	0.730629	0.557804	0.868306
Folded Normal	0.574161	0.998554	0.735692

* K-S; Kolmogorov–Smirnov; ** A-D; Anderson–Darling.

Table S5. Non-parametric data distribution fits—release profiles of amylopectin formulations.

	K-S *p*-Value		
**Distribution**	**Redispersed**	**Dried**	**Emulsions**
Gaussian Mixture	0.999819	0.999917	0.999984
Johnson SB	0.999969	0.999940	0.999826
Half Normal	0.999696	0.999711	0.998795
Weibull	0.997181	0.997524	0.997543
General Extreme Value	0.999003	0.998568	0.998693
Log Normal	0.999770	0.999400	0.999652
Normal	0.919261	0.923058	0.939447
General Pareto	0.674234	0.726308	0.868306
Triangular	0.913416	0.862547	0.888420
Rayleigh	0.511783	0.557804	0.735692
Folded Normal	---	0.998554	---

* K-S; Kolmogorov–Smirnov; ** A–D; Anderson–Darling.

Table S6. Non-parametric data distribution fits—release profiles of alginic acid formulations.

	K-S *p*-Value		
**Distribution**	**Redispersed**	**Dried**	**Emulsions**
Gaussian Mixture	0.999959	0.999946	0.999931
Johnson SB	0.999982	0.999961	0.999911
Half Normal	0.999720	0.999730	0.998134
Weibull	0.999370	0.998903	0.990192
General Extreme Value	0.998873	0.999002	0.997881
Log Normal	0.990871	0.999601	0.999872
Normal	0.953931	0.934529	0.903009
General Pareto	0.805552	0.750744	0.721534
Triangular	0.872663	0.897271	0.854497
Rayleigh	0.642984	0.591398	0.721534
Folded Normal	---	---	---

* K-S; Kolmogorov–Smirnov; ** A-D; Anderson–Darling.

S3.Cumulative Release

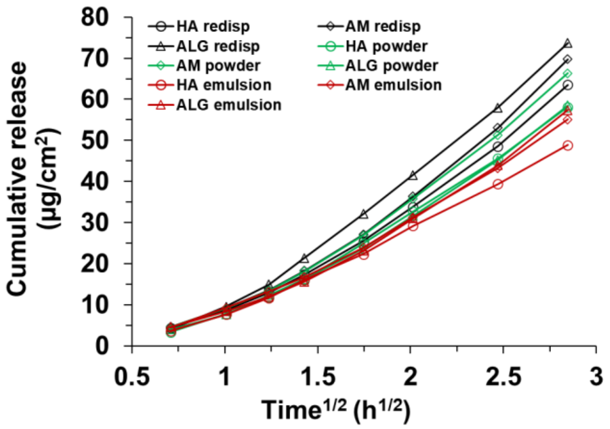

Figure S6. All cumulative release curves of griseofulvin from all the investigated formulations.

The emulsions are shown as red curves, the dry emulsion powders are shown as green curves, and the redispersed emulsions are shown as black curves.
